# OA08 The dangers of ‘therapeutic nihilism’ in anti-phospholipid syndrome

**DOI:** 10.1093/rap/rkag113.008

**Published:** 2026-11-03

**Authors:** Jyothi Sivasothy, Thiraupathy Marianayagam, Spencer Ellis

**Affiliations:** East and North Hertfordshire NHS Trust, Stevenage, United Kingdom; East and North Hertfordshire NHS Trust, Stevenage, United Kingdom; East and North Hertfordshire NHS Trust, Stevenage, United Kingdom

## Abstract

**Introduction:**

Antiphospholipid syndrome (APS) is an autoimmune thrombotic disorder characterised by recurrent venous or arterial thrombosis in the presence of antiphospholipid antibodies. Catastrophic antiphospholipid syndrome (CAPS) is a rare but life-threatening sub-type, causing extensive microvascular thrombosis and subsequent multi-organ failure. Due to its rarity and clinical overlap with sepsis and other causes of organ dysfunction, diagnosis can be challenging. We present a case of APS progressing to CAPS with severe multi-organ failure. This case demonstrates the value of diagnostic vigilance in identifying reversible causes of multi-organ failure, with early specialist intervention at a tertiary centre leading to marked clinical recovery.

**Case description:**

A 47-year-old woman with longstanding autoimmune rheumatic disease, predominantly characterised by Raynaud’s phenomenon, was not under regular follow-up. She presented with drowsiness, cyanotic fingertips, toes, and nose with lower limb swelling. Initial investigations demonstrated multi-organ dysfunction, including acute kidney injury (AKI), deranged liver function tests, thrombocytopenia, and anaemia. Autoimmune screening revealed positive ANA and anti-dsDNA antibodies with hypocomplementemia.

Her condition rapidly deteriorated with worsening confusion, requiring transfer to critical care. She was initially treated for lupus cerebritis. MRI brain demonstrated multiple acute ischaemic infarcts. In the context of progressive multi-organ involvement, CAPS was suspected. Antiphospholipid screen was triple positive with high titre IgG anti-cardiolipin antibodies of 124U/ml. AKI was apparent with creatinine peak of 192umol/l. She was managed with cyclophosphamide followed by plasma exchange, IVIG and subsequently rituximab.

NT-proBNP was elevated at 12880ng/l.  Echocardiography demonstrated a rheumatic-appearing mitral valve with severe mitral regurgitation, moderate mitral stenosis, and features suggestive of Libman–Sacks endocarditis. Her admission was further complicated by an abdominal wall haemorrhage requiring blood transfusion. Therapeutic enoxaparin was stopped and restarted on multiple occasions due to recurrent bleeding concerns. Given the extensive thrombotic burden and ongoing concern for CAPS, anticoagulation was ultimately continued.

Serial echocardiography demonstrated progressive deterioration in mitral valve function. Local cardiology review deemed her unsuitable for percutaneous intervention. Withdrawal of active treatment was discussed with the family due to perceived poor prognosis. The rheumatology team requested a second opinion at a tertiary cardiology centre and the patient was accepted. Immediate mitral valve intervention was not feasible but tertiary input enabled optimisation of treatment.

Despite presenting with severe multi-organ failure and an initially grave prognosis, the patient achieved a delayed but significant recovery. She was transferred back to her local hospital for rehabilitation and rheumatology follow-up, demonstrating the potential for reversibility in severe CAPS.

**Discussion:**

CAPS is a rare, life-threatening thrombotic disorder associated with significant mortality due to rapid progression to multi-organ failure. The severity of presentation, however, should not be interpreted as evidence of irreversible disease. This case demonstrates the dangers of ‘therapeutic nihilism’ in patients with severe CAPS.

Despite widespread neurological involvement with multifocal cerebral infarction, hepatic dysfunction, acute kidney injury, significant valvular disease, and haematological abnormalities, our patient demonstrated substantial recovery following appropriate immunosuppressive approaches and anticoagulation. Referral to a tertiary centre was essential in allowing continued reassessment of cardiac treatment options and ultimately altered the patient’s clinical trajectory.

An important consideration in the management of CAPS is that clinical improvement may not be instant. Given the severity of organ injury and the lag time for rituximab response, a lack of early improvement should not be interpreted as treatment failure. Decisions regarding withdrawal of therapy or limitations of care should therefore be made cautiously. In this case, perseverance with treatment despite ongoing multi-organ dysfunction was followed by recovery of renal function, improvement in cognitive status, and progression to stroke rehabilitation.

This case also demonstrates the complex balance between thrombosis and haemorrhage in CAPS. The patient developed a sizeable intra-abdominal wall haemorrhage requiring blood transfusion; however, anticoagulation was ultimately continued because the underlying pathophysiology of CAPS is driven by uncontrolled thrombosis. The decision to continue enoxaparin despite bleeding risk required careful multidisciplinary discussion between haematology, rheumatology, and critical care teams, balancing the risk of further haemorrhage against progression of thrombotic disease.

Overall, this case demonstrates that severe multi-organ failure in CAPS should not preclude escalation of care. Timely specialist input, persistence with therapy, and careful multidisciplinary decision-making can result in meaningful recovery despite an initially grave presentation.

**Key learning points:**

• CAPS should be considered in patients presenting with rapidly progressive multi-organ dysfunction, particularly when there is evidence of thrombosis affecting multiple vascular territories and underlying autoimmune disease.

• Early recognition and prompt initiation of aggressive treatment, including anticoagulation, immunosuppression, and plasma exchange, are essential to prevent ongoing thrombotic injury and improve outcomes.

• Clinical improvement in CAPS may be delayed, particularly in patients with extensive organ dysfunction; a lack of immediate response should not be perceived as treatment failure, and decisions to withdraw or limit therapy should be made cautiously. Recovery may occur gradually following initiation of immunosuppression, plasma exchange, anticoagulation and significant stroke rehabilitation.

• Severe multi-organ failure should not lead to ‘therapeutic nihilism’ or assumptions that disease is irreversible. Although the presence of neurological, renal, cardiac, and haematological involvement is associated with a high burden of morbidity and mortality, this case demonstrates that meaningful functional and physiological recovery remains possible. Aggressive, timely, and sustained treatment, supported by close multidisciplinary management, may result in favourable outcomes even in patients presenting with profound systemic involvement.

• Early transfer to a tertiary centre should not be delayed on the basis of disease severity alone. Specialist multidisciplinary input can provide diagnostic clarification, guide complex treatment decisions, and alter clinical outcomes.

• The management of CAPS requires careful balancing of thrombosis and haemorrhage. Significant bleeding complications may necessitate repeated reassessment of anticoagulation, but anticoagulation may still need to be continued due to the high risk of ongoing catastrophic thrombosis. Failing to manage this ultimately leads to rapidly progressive CAPS with worsening thrombotic manifestation.

